# Case report: A case of pseudo-acute kidney injury due to cyclin-dependent kinase inhibitor

**DOI:** 10.3389/fneph.2024.1389562

**Published:** 2024-06-10

**Authors:** Praveen Errabelli, Maulik Lathiya, Devender Singh

**Affiliations:** ^1^ Department of Nephrology and Hypertension, Mayo Clinic Health System, Eau Claire, WI, United States; ^2^ University of Minnesota Health Sciences, University of Minnesota Medical Center, Minneapolis, MN, United States

**Keywords:** acute kidney injury, pseudo acute kidney injury, CDK (cyclin-dependent kinase), cystatin C (Cys C), EGFR, creatinine clearance, abemaciclib

## Abstract

Various classes of targeted therapies have emerged in the last few years, which have revolutionized cancer treatment, and improved the prognosis and survival of cancer patients. Unfortunately, these agents have serious toxic effects on the kidneys. Some of the toxic effects are hypertension, acute kidney injury (AKI), and proteinuria. One interesting phenomenon that has emerged recently is pseudo-acute kidney injury due to the interference with the tubular secretion of creatinine by some of the targeted therapeutic agents. Understanding this physiology is needed to avoid unnecessary investigation and withholding of lifesaving chemo regimen. Alternative methods to assess renal function such as cystatin C-based estimated glomerular filtration rate (eGFR) can differentiate true AKI from pseudo-AKI. Here, we describe one such case of pseudo-AKI from cyclin-dependent kinase (CDK) 4/6 inhibitor, abemaciclib, which inhibits tubular secretion of creatinine. Using cystatin-C-based eGFR revealed pseudo-AKI in this case.

## Introduction

Significant progress has been made when it comes to targeted cancer therapies in recent years. These newer agents pose new challenges to nephrologists due to their various adverse effects on the kidneys. Knowledge about various adverse effects associated with each agent is essential as the lack of in-depth understanding of the underlying pathophysiology of these effects can lead to untimely discontinuation of much-needed cancer therapy. Here, we present a case of suspected nephrotoxicity associated with abemaciclib (CDK4/6 inhibitor), which turned out to be a pseudo-acute kidney injury due to its interference with the tubular secretion of creatinine. The purpose of reporting this case is to increase awareness among clinicians about this entity, which would help avoid unnecessary investigations, delays in treatment, and inadvertent discontinuation of chemotherapeutic agents.

## Case description

**Figure 1:**
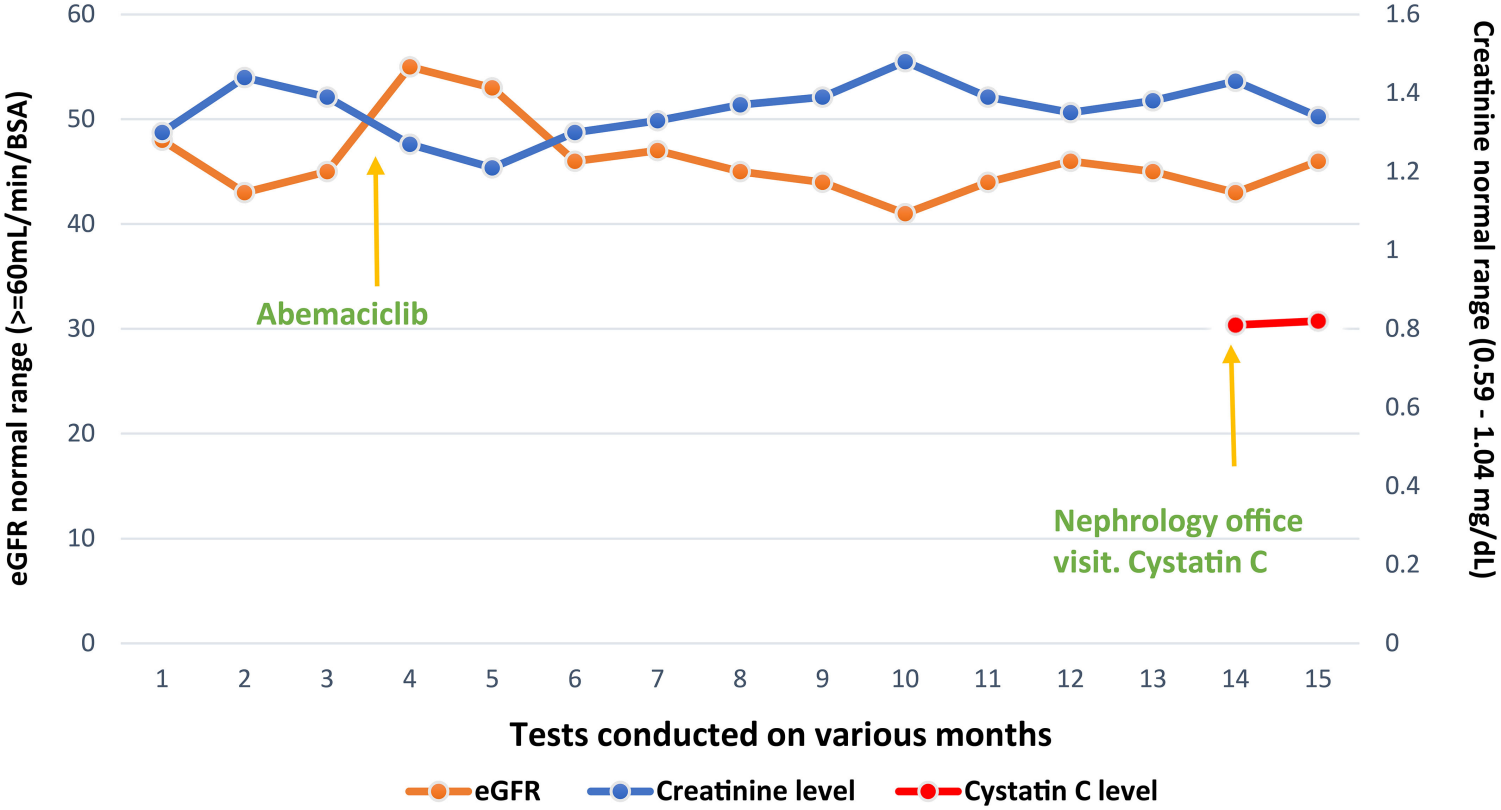
Serum creatinine-based eGFR and serum cystatin C.

A 55-year-old postmenopausal female patient was referred by an oncologist for declining renal function. She was diagnosed with invasive ductal carcinoma of the left breast, stage II A, ERPR positive, HER2/neu negative, cT1c N1 M0. She underwent neoadjuvant chemotherapy with four cycles of docetaxel/cyclophosphamide, left lumpectomy, and axillary node dissection. Despite a seemingly positive response to neoadjuvant treatment, posttreatment MRI showed a significant residual disease and the final pathology report revealed pT1c N1 disease with 2/12 lymph nodes positive for carcinoma, one lymph node showing extranodal extension. The treatment plan included adjuvant radiation therapy and Arimidex 1 mg daily for 10 years, and adjuvant abemaciclib 150 mg twice a day for 2 years. Prior to starting abemaciclib, the patient had a baseline plasma creatinine of 0.8–0.9 mg/dL with a creatinine-based eGFR of 80 mL/min. However, a month after initiation of abemaciclib, creatinine rose to 1.13 mg/dL and creatinine-based eGFR dropped to 45 mL/min. A month later, plasma creatinine remained elevated at approximately 1.2–1.4 mg/dl (**Figure 1**), prompting nephrology referral.

During the visit to a nephrology clinic, the patient denied any shortness of breath, chest pain, palpitation, vomiting, pain abdomen, dysuria, hematuria, foamy urine, increased frequency of urination, lower extremity swelling, and skin rash. She had no prior history of kidney disease but she had fatigue, night sweats, stiffness in the joints, numbness in hands and feet, and headaches. She did report taking ibuprofen 600 mg intermittently for at least 1–2 years to manage headaches, which she had stopped taking 2 months prior to nephrology visit. Her blood pressure was 121/83 mm/hg, pulse rate 68 beats/min, and the rest of the physical examination was normal.

Laboratory tests including urinalysis, 24-h urine protein, cystatin C-based eGFR, basic metabolic panel, and 24-h creatinine clearance were done. Urine sediment did not show any hematuria, pyuria, or proteinuria. Cystatin C-based eGFR was 95 mL/min/BSA and 97 mL/min/BSA (checked twice 1 month apart). The 24-h urine creatinine clearance was 67 mL/min/BSA and the 24-h urine protein was only 115 mg (**Table 1**).

**Table 1:** Laboratory results.

Parameter	Value		Unit
24-h urine protein	115		mg
Creatinine clearance	67		mL/min
24-h urine volume	2,300		mL
Creatinine	59		mg/dL
Plasma creatinine	1.37		mg/dL
**Test**	**17/07/2023**	**14/08/2023**	**18/08/2023**
Cystatin C	0.82	0.81	
Cystatin C eGFR	95	97	
Sodium, P	140	139	140
Potassium, P	4.5	4.2	4.4
Chloride, P	105	102	102
Bicarbonate, P	26	27	26
Anion gap, P	9	10	12
Blood urea nitrogen (BUN), P	16	15	16
Creatinine	1.38 (H)	1.43 (H)	1.34 (H)
Estimated GFR (eGFR)	45 (L)	43 (L)	46 (L)
Calcium, total, P	9.5	9.1	9.6
Glucose, P	91	65 (L)	91
Phosphorus (inorganic), P	3.2		3.5
Albumin, P	4.5	4.0	4.4
Bilirubin, total, P		0.3	
Bilirubin, direct, P		<0.2	
Alanine aminotransferase (ALT), P		16	
Aspartate aminotransferase (AST), P		22	
Alkaline phosphatase, P		33 (L)	
Protein, total, P		6.3	
**Parameter**		**17/07/2023**	
Color, U		Yellow	
Clarity		Clear	
Source		Urine, midstream	
Nitrite, U		Negative	
Leukocyte esterase		Negative	
pH		7.5	
Specific gravity		1.005	
Glucose		Negative	
Protein urine random		Negative	
Ketone		Negative	
Bilirubin		Negative	
Blood		Negative	
Urobilinogen		0.2	
White blood cells		None seen	
Red blood cells in urine		None seen	

(H, high; L, low).

Since cystatin C-based eGFR was much higher than creatinine-based eGFR and renal function was stable, it was determined that we were dealing with pseudo-AKI due to reduced creatinine secretion with the use of abemaciclib. This is also confirmed by low 24-h creatinine clearance when compared to cystatin C-based eGFR.

After this determination, we recommended that abemaciclib be continued and cystatin C-based eGFR with regular Basic Metabolic panel (BMP) be checked. The patient continues to have stable renal function despite being on abemaciclib for more than a year (**Figure 1**).

## Discussion

The advent of targeted therapies has revolutionized cancer care and enhanced outcomes. Nonetheless, their nephrotoxic properties have introduced new challenges to nephrology. These therapies have the potential to induce renal damage by targeting molecules like vascular endothelial growth factor and platelet-derived growth factor, which are expressed in normal nephrons (1). The predominant adverse event is acute kidney injury, which may manifest as acute tubular necrosis, acute interstitial nephritis, or glomerular injury leading to hematuria and proteinuria. This condition is diagnosed by monitoring the patient’s serum creatinine levels, creatinine-based eGFR, urine sediment for hematuria and proteinuria, and, in certain cases, performing a kidney biopsy. Additionally, a new entity termed pseudo-AKI has recently emerged. This is attributed to the disruption of the tubular secretion of serum creatinine by certain targeted therapies. Some of these targeted therapies interfere with the tubular secretion of serum creatinine by affecting the activity of organic cation transporter (OCT) and multidrug and toxic compound extrusion (MATE) transporters. Specifically, the facilitation of creatinine secretion from proximal tubules is mediated by organic cation transporter 2 (OCT2) and multidrug and toxin compound extrusion protein 1 (MATE1) (2).

The way to differentiate pseudo-AKI from true AKI is by measuring serum cystatin C-based eGFR along with serum creatinine-based eGFR and using alternative clearance estimation methods like iothalamate clearance. Various targeted therapies, including anaplastic lymphoma kinase (ALK) inhibitors, mesenchymal epithelial transition (MET) inhibitors, cyclin-dependent kinase (CDK4/6) inhibitors, poly ADP-ribose polymerase inhibitors (PARP), v-raf murine sarcoma viral oncogene homolog B1 (BRAF) inhibitors, Breakpoint Cluster Region–v-Abl Abelson murine leukemia viral oncogene homolog (BCR-ABL) inhibitors, HER-2 inhibitors, and tyrosine kinase inhibitors, have been associated with pseudo-AKI. If the patient has true AKI, treatment should be withheld or modified to avoid further complications. However, if pseudo-AKI is identified, discontinuing chemotherapy can be avoided, potentially benefiting the patient (1).

Cyclin-dependent kinases 2/4/6 (CDK 2/4/6) control the progression of cell cycle checkpoints from G1 to S phase (3). CDK4/6 inhibitors promote G0/G1 arrest in renal epithelial cells (3). The combination of a cyclin-dependent kinase (CDK) 4/6 inhibitor with an aromatase inhibitor represents the primary therapy for hormone receptor (HR)-positive, human epidermal growth factor receptor 2 (HER2)-negative metastatic breast cancer (1). CDK 4/6 inhibitors block renal transporters such as organic cation transporter 2 (OCT 2), MATE1, and MATE-2K, resulting in decreased creatinine secretion into the renal tubule. This mechanism results in elevated creatinine levels and should not be interpreted as indicative of acute kidney injury (2).

Abemaciclib, an oral chemotherapeutic medication, is indicated for the treatment of metastatic breast cancer. Abemaciclib was approved as adjuvant therapy for the treatment of high-risk hormone receptor-positive, HER2 negative breast cancer based on MONARCH 1, 2, and 3 trials. The trials demonstrated that the addition of abemaciclib led to a significant and clinically meaningful improvement in invasive disease-free survival in the intention-to-treat population compared with endocrine therapy alone (4–6).

It acts as a potent and selective inhibitor of cyclin-dependent kinases 4 and 6 (7). Abemaciclib is known to inhibit OCT2, MATE1, and MATE2-K. Clinically assessed parameters such as glomerular filtration rate, serum cystatin-C levels, serum neutrophil gelatinase-associated lipocalin (NGAL), and urine markers of kidney tubular injury, such as kidney injury molecule-1, are minimally affected by abemaciclib (7). These findings suggest that the observed elevations in serum creatinine, commonly utilized to estimate glomerular filtration rate (creatinine-based eGFR), are likely attributable to the suppression of proximal tubule secretory transporters (7). Abemaciclib treatment may lead to a slight (approximately 10%–40%) reversible increase in serum creatinine in patients. However, for accurate assessment of renal function in abemaciclib-treated patients, alternative methods for evaluating renal function should be employed as needed (7). The active transport of creatinine within renal tubules involves various transport proteins, such as OCT2, MATE1, and MATE2‐K. OCT2 facilitates creatinine uptake from the blood into proximal tubule cells via facilitated diffusion at the basolateral membrane, while MATE1 and MATE2‐K, located at the apical membrane, promote creatinine and other compound efflux from cells into urine through proton-coupled transport. The inhibition of OCT2 and MATEs reduces creatinine clearance, leading to elevated serum creatinine levels due to impaired tubular secretion. These alterations may occur without significant changes in renal function or measured glomerular filtration rate (GFR) (7). Alternative markers like cystatin C offer a dependable approach for assessing GFR and renal function, as they are not influenced by active secretion and remain unaffected by dietary variations or changes in muscle mass (7). Cystatin C, a non-glycosylated protein weighing 13.3 kDa, is part of the cystatin superfamily of cysteine protease inhibitors. It acts as a pro-survival protein in cells under stress conditions. Being entirely catabolized in the proximal renal tubule without reabsorption into circulation following glomerular filtration, cystatin C serves as an ideal indicator for estimating GFR. Its measurement is less influenced by factors like age, gender, muscle mass, and ethnicity under normal conditions, making it particularly valuable for early therapeutic interventions. Consequently, cystatin C emerges as an excellent marker for estimating GFR, especially in the initial therapeutic stages and during patient monitoring (8).

Clearance rates of exogenous compounds like iohexol, which are freely filtered and not metabolized, provide a reliable method for accurately determining absolute glomerular filtration rate (GFR) and offering a more precise assessment of glomerular filtration activity. Several biomarkers, including kidney injury molecule 1 (KIM‐1) and NGAL, show potential in identifying acute kidney injury compared to serum creatinine (7). Notably, although the creatinine-based GFR [via Chronic Kidney Disease Epidemiology Collaboration (CKD-EPI)] decreased following abemaciclib administration, this decline was not reflected in the measured GFR determined by iohexol clearance or GFR calculated from serum cystatin‐C concentrations. Furthermore, significant changes were not observed in the levels of NGAL and KIM‐1 in urine normalized to creatinine, both indicative of renal injury. These findings suggest that the observed increases in serum creatinine post-abemaciclib administration stem from reversible inhibition of renal tubular creatinine secretion rather than acute kidney injury (7). Therefore, the impact of abemaciclib on renal function, as assessed by measured GFR and urinary biomarkers of renal injury, appears minimal. Patients receiving abemaciclib may experience a mild and reversible (~10%–40%) elevation in serum creatinine due to inhibition of renal transport, highlighting the need for alternative renal function assessment methods when clinically indicated (7). Similar observations were made during MONARCH 1, 2, and 3 trials (4–6). In practical terms, the measurement of cystatin C-based estimated GFR, which remains unaffected by medications inhibiting creatinine secretion, may assist in distinguishing nephrotoxicity from pseudo-acute kidney injury (9). It is important to note that factors such as inflammation, thyroid dysfunction, and smoking influence cystatin C levels (10, 11).

The primary adverse effects linked with CDK4/6 inhibitors include neutropenia, leukopenia, and fatigue, although acute kidney injury is rarely observed (12). Distinguishing between pseudo-acute kidney injury (AKI) and true AKI presents a challenge. Simultaneous evaluation of serum creatinine, kidney iothalamate clearance, and estimated glomerular filtration rate (eGFR) based on cystatin C levels assists in distinguishing between these conditions. Recent eGFR equations that incorporate both creatinine and cystatin C levels, without considering race, provide more accurate estimations of measured GFR compared to formulas utilizing either marker independently. This results in reduced differences in measured GFR across various racial demographics (13, 14).

Various drugs known to inhibit the tubular secretion of creatinine are summarized in **Table 2** (15–17).

**Table 2:** Summary of medications known to inhibit tubular secretion of creatinine leading to a rise in plasma creatinine.

Drugs	Mechanism of action	Uses
Cimetidine, famotidine, and ranitidine	Histamine 2 receptor blocker	Antacid
Trimethoprim	Folate synthesis inhibitor	Antibiotic
Pyrimethamine	Dihydrofolate inhibitor	Malaria and toxoplasma
Salicylates	Cyclooxygenase inhibitors	Anti-inflammatory and antiplatelet
Cobicistat	Cytochrome P450 (CYP) 3A inhibitor	HIV infection
Calcitriol	Synthetic vitamin D analog	Osteoporosis and hypocalcemia
Abemaciclib, ribociclib, and palbociclib	CDK 4/6 inhibitors	Metastatic HR-positive and HER2-negative breast cancer
Olaparib, niraparib, and rucaparib	PARP Inhibitors	Ovarian cancer (BRCA1/2 +), metastatic breast cancer (BRCA1/2 +), and endometrial cancer
Imatinib, crizotinib, alectinib, ceritinib, gefitinib, pazopanib, sunitinib, sorafenib, and tucatinib	Tyrosine kinase inhibitors	NSCLC, CML, ALL, GIST, RCC, soft tissue sarcomas, GIST, HCC, thyroid cancer, and advanced or metastatic HER2-positive breast cancer
Capmatinib	MET inhibitors	NSCLC

CDK, cyclin-dependent kinase; HR, hormone receptor; HER2, human epidermal growth factor receptor 2; PARP, poly(adenosine diphosphate-ribose) polymerase; NSCLC, non-small cell lung cancer; CML, chronic myelogenous leukemia; ALL, acute lymphocytic leukemia; GIST, gastrointestinal stromal tumor; RCC, renal cell carcinoma; HCC, hepatocellular carcinoma; and MET, mesenchymal-epithelial transition.

## Conclusion

In our patient’s case, serum creatinine was elevated, and the creatinine-based eGFR has declined after starting on abemaciclib. However, after checking cystatin C levels and cystatin C-based eGFR, it was confirmed that the patient was having pseudo-AKI due to inhibition of creatinine secretion. This in turn has helped us reassure the patient and our oncology colleagues that the patient does not have any true kidney injury from abemaciclib and that chemotherapy can be continued. Our goal in presenting this article is to create awareness among clinicians about pseudo-AKI in patients receiving CDK inhibitors and the importance of checking alternate markers of estimating renal function like cystatin C.

## Data availability statement

The original contributions presented in the study are included in the article/supplementary material. Further inquiries can be directed to the corresponding author.

## Ethics statement

Written informed consent was obtained from the individual(s) for the publication of any potentially identifiable images or data included in this article. Written informed consent was obtained from the patient for writing up and publishing this case report.

## Author contributions

PE: Conceptualization, Investigation, Supervision, Writing – original draft, Writing – review & editing. ML: Data curation, Writing – review & editing. DS: Investigation, Writing – review & editing.
